# ngx-mol-viewers: Angular components for interactive molecular visualization in bioinformatics

**DOI:** 10.3389/fbinf.2025.1586744

**Published:** 2025-06-26

**Authors:** Damiano Clementel, Alessio Del Conte, Alexander Miguel Monzon, Silvio C. E. Tosatto

**Affiliations:** ^1^ Department of Biomedical Sciences, University of Padova, Padova, Italy; ^2^ European Molecular Biology Laboratory, European Bioinformatics Institute (EMBL-EBI), Wellcome Genome Campus, Hinxton, Cambridgeshire, United Kingdom; ^3^ Institute of Biomembranes, Bioenergetics and Molecular Biotechnologies, National Research Council (CNR-IBIOM), Bari, Italy

**Keywords:** bioinformatics, Angular, web components, biological visualization, typescript

## Abstract

Advancements in bioinformatics have been propelled by technologies like machine learning and have resulted in substantial increases in data generated from both empirical observations and computational models. Hence, well-known biological databases are growing in size and centrality by integrating data from different sources. While the primary goal of these databases is to collect and distribute data through application programming interfaces (APIs), providing visualization and analysis tools directly on the browser interface is crucial for users to understand the data, which increases the usefulness and overall impact of the databases. Currently, some front-end frameworks are available for the sustained development of the user interface (UI) and user experience (UX) of these resources. Angular is one of the most popular frameworks to be broadly adopted within the BioCompUP laboratory. This work describes a library of reusable and customizable components that can be easily integrated into the Angular framework to provide visualizations of various aspects of protein molecules, such as their sequences, structures, and annotations. Currently, the library includes three main independent components. The first is the ngx-structure-viewer, which allows visualization of molecules through the MolStar three-dimensional viewer. The second is the ngx-sequence-viewer, which provides visualization and annotation capabilities for a single sequence or multiple sequence alignments. The third the ngx-features-viewer, enables the mapping and visualization of various biological annotations onto the same molecule. All these tools are available for download through the Node Package Manager (NPM), and more information is available at https://biocomputingup.github.io/ngx-mol-viewers/ (under development).

## Introduction

There has been a growing interest in bioinformatics in recent years due to advances in personalized medicine, biological data analysis capabilities, and protein structure prediction (Sehnal et al., 2021).

 Biological databases continue to expand rapidly due to technological advancements that enable faster and cheaper biological observations. Machine learning tools such as AlphaFold (Jumper et al., 2021) and RoseTTAFold (Baek et al., 2021) have been successfully used to predict protein structures with high accuracy. The increase in publicly available data has enhanced our understanding of biological phenomena, but often requires database reengineering for scalability. This has become necessary for databases such as MobiDB (Piovesan et al., 2025) and RepeatsDB (Clementel et al., 2025).

The growth of data in biological databases presents challenges not just for storage but also for user interactions. As databases expand, visual exploration tools become essential as users cannot feasibly download large datasets to local machines. Hence, modern databases often provide integrated visualization tools that allow researchers to explore data directly within the interface, including features to navigate between sequences and structures, map annotations onto 3D models, and link different information layers.

 Web technologies are the most common choice for accessibility, as they require only a browser and an internet connection, without additional software installation. Front-end frameworks have emerged as a popular choice for creating highly interactive web applications capable of handling complex tasks. Despite not being the most popular framework (Technology, 2024), the BioComputing UP laboratory at the University of Padova selected Angular (2025) as the primary framework for developing front-end interfaces for their biological databases, such as MobiDB, RepeatsDB, DisProt (Aspromonte et al., 2024), and PED (Ghafouri et al., 2024), along with web services such as RING (Clementel et al., 2022; Del Conte et al., 2024), making it a requirement for this project.

**FIGURE 1:**
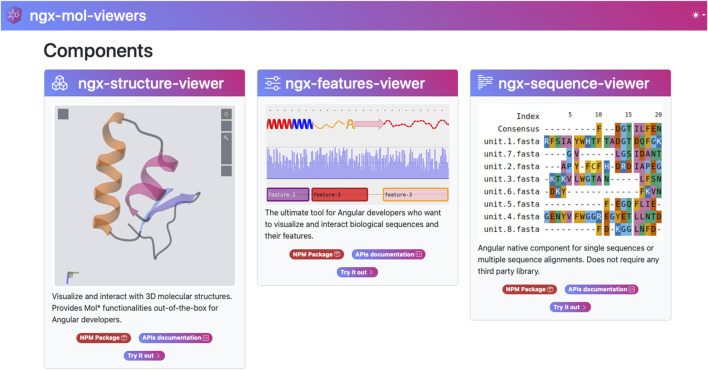
The three main components of the ngx-mol-viewers library: the ngx-structure-viewer, the ngx-features-viewer, and the ngx-sequence-viewer, as shown on the project homepage (https://biocomputingup.github.io/ngx-mol-viewers/). Each component has a dedicated node package manager (NPM) repository and a page on the project website.

Although developed as separate projects, all of the above databases use shared visualization tools for biological molecules and are built on the Angular framework. This resulted in the development of an Angular-based library containing generic tools for visualizing biological molecule information from various perspectives (Figure 1). Although these tools were initially conceived to reduce development effort and improve maintainability within existing projects, publishing them as open-source Node Package Manager (NPM) (npmjs, 2025) components is expected to allow the broader bioinformatics community that uses Angular to benefit from them.

## Methods

The ngx-mol-viewers library includes three Angular components: the structure viewer, the features viewer, and the sequence viewer. All of these components are built as standalone components in Angular and published on NPM separately. Therefore, they can easily be installed using NPM in any Node.js (2025) project using the commands shown in Algorithm 1:

Algorithm 1 Bash commands used to install the components in the ngx-mol-viewers library in a given Node.js project using NPM.

1 npm install ngx-structure-viewer
2 npm install ngx-features-viewer
3 npm install ngx-sequence-viewer

Then, these packages can be imported into Angular using the import notation in the corresponding TypeScript files. Algorithm 2 shows an example for importing the ngx-sequence-viewer component. This imported component will then be available in the Angular HTML template, as shown in Algorithm 3.

Algorithm 2 TypeScript example for importing the ngx-sequence-viewer component into Angular.

1 **import** { Ngx Sequence Viewer Component } **from** (′@ngx-sequence-viewer′ ;
2 
3 @Component ({
4  imports : [ Ngx Sequence Viewer Component ],
5 })
6 **export class** My Component {}

Algorithm 3 HTML template of the imported ngx-sequence-viewer component in Angular. The inputs are defined in lines 3–6 using square brackets, and the output is shown in line 8 bound to the on Selected method of the importing component in the parentheses.

1 <ngx-sequence-viewer

2 <!-- Set input --!>

3 [ sequence ]= “sequence”

4 [ index ]= “index”

5 [ loci ]= “loci”

6  [settings]=“settings$ | async”
7 <!--Bind output--!>

8  (selected$)=“onSelected($event)”
9 > </ngx-sequence-viewer>

The state of a component can be changed using the input properties, which are clearly defined within the Angular framework as the preferred method of parsing data from the importing parent component to the imported child component. Among the inputs that can be set, there are definitions for the target entity to be visualized as annotations for the same entity, and overall representation settings, which are common to the three package components described in this work. Examples of these inputs are shown in lines 3–6 of Algorithm 3. In line 6 of Algorithm 3, the settings are assigned using an asynchronous pipeline as RxJS (2025) observables, which dynamically change values according to user and browser behaviors. The components in the ngx-mol-viewers library can handle these changes out of the box without reinitialization.

The outputs defined by the components collected in the ngx-mol-viewers library are RxJS observables. These outputs are defined within the Angular framework as the preferred mode of parsing data from imported child components to importing parent components. Thus, they can be used to bind a method in the importing component, allowing data to flow from imported components to importing components and then to other imported components within the same package. The similarity between the data structures in the components of the ngx-mol-viewers library allows for easy interaction among them.

Line 8 of Algorithm 3 shows an example of an output defined in the ngx-sequence-viewer, i.e., *selected$*. In this case, the output is triggered each time a user selects one or more contiguous positions in the sequence viewer instance using the drag-and-drop gesture. It should be noted that the dollar notation used to define the *selected$* output name is commonly associated with RxJS observables and must not be confused with the dollar notation used in $event, which is the value parsed by Angular to the output method. For more detailed information, the project website is available at https://github.com/BioComputingUP/ngx-mol-viewers.

## Results

### ngx-structure-viewer

**FIGURE 2:**
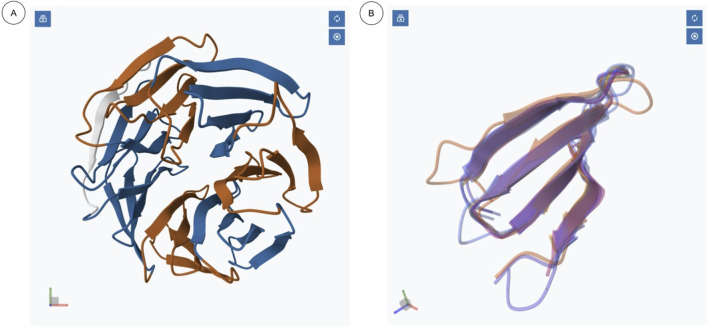
Example of two ngx-structure-viewer instances used on the same page for the structure with the PDB code: 5eam chain A, as represented in RepeatsDB: **(A)** repeated units annotated on the structure; **(B)** repeated units in the same structure, as returned by the multiple structural alignment software mTm-Align (Dong et al., 2018) and shown as overlaps. Ngx-structure-viewer allows setting an alpha value of less than 1 to allow better representation of densities.

The ngx-structure-viewer is built upon the MolStar viewer library, which provides three-dimensional visualization of molecular complexes (Figure 2). MolStar is widely used by several bioinformatics resources, including PDBe (Armstrong et al., 2019), UniProtKB (The UniProt Consortium et al., 2025), AlphaFoldDB (Varadi et al., 2022), and InterPro (Blum et al., 2025). This component encapsulates a MolStar instance within the Angular lifecycle to ensure effortless integration into Angular projects. It allows users to define the molecular complex to be visualized through an Angular input, thus providing flexibility to select and customize the displayed structure.

Additional inputs enable modification of the representations of specific sections of the molecule and visualization of molecular contacts within the complex. The component also intercepts essential user interactions, such as selecting and hovering over entities within the molecular complex. These interactions are exposed via Angular outputs to facilitate seamless communication with other Angular components.

### ngx-features-viewer

**FIGURE 3:**
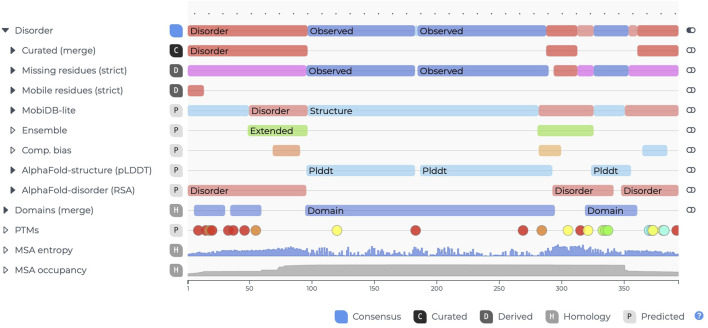
Example of the ngx-features-viewer for entry P04637 (cellular tumor antigen) in MobiDB. The filled black carets, such as the one next to “Disorder,” indicate nested annotations that can be revealed by clicking. When expanded, the carets point downward.

The ngx-features-viewer (Figure 3) builds on the experience gained from the feature-viewer library while drawing inspiration from other non-Angular projects, such as Nightingale (Salazar et al., 2023). It represents a comprehensive reengineering of the original feature-viewer library (Paladin et al., 2020) by leveraging the D3 library (D3 by Observable, 2025) to achieve full Angular lifecycle compliance.

The key features include simplified integration into Angular projects, as no additional overhead is needed to manage the D3 library;

• enhanced customization options, including adjustable labels and tooltips, by exploiting content projection; improved vertical axis alignment to address issues present in the original feature-viewer; and optimization for large-scale, deeply nested annotations that are tailored to the requirements of databases like MobiDB. This component allows users to efficiently visualize sequence features, making it highly suitable for applications requiring detailed annotation displays.

### ngx-sequence-viewer

**FIGURE 4:**
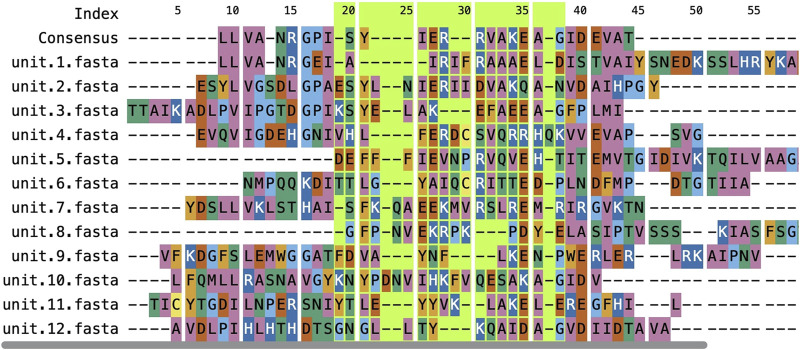
Example of the ngx-sequence-viewer showing a multiple sequence alignment for UniProtKB entry A0A0H3JRU9 in RepeatsDB. The component visualizes a FASTA-formatted alignment retrieved by sequential alignment of repeated units using the Clustal Omega software (Sievers and Higgins, 2018). The region with the green background was set by dragging the mouse cursor over the component, and the boundaries of such regions are obtained using an output observable.

The ngx-sequence-viewer (Figure 4) was developed to address the integration overhead experienced with ProSeqViewer (Bevilacqua et al., 2022) when used in Angular projects. This component removes the complexity of embedding sequence viewers within Angular, making the process more straightforward and efficient.

The key functionalities here include the ability to accept either a single sequence or a multiple sequence alignment as input, customizable visualization of subsections with distinct color schemes, and the ability to expose selection events as Angular outputs to enable data sharing and interactivity with other components. This design enhances the user experience and promotes inter-component communication in complex Angular applications.

## Discussion

The ngx-mol-viewers library provides a comprehensive suite of tools for molecular visualization within Angular applications. When these tools are implemented as Angular standalone components, they are agnostic to the application in which they are used, ensuring seamless integration and enhanced interactivity in modern bioinformatics web applications. Although they draw inspiration from previously published components, they are unique in that they are fully compliant with the Angular lifecycle, which allows out-of-the-box usage once imported into an Angular project.

The library currently includes three main components:

• ngx-structure-viewer, for rendering and interacting with molecular structures,
• ngx-features-viewer, for visualizing annotated features along sequences, and
• ngx-sequence-viewer, for exploring biological sequences directly.

These components are designed to be generic and adaptable across multiple bioinformatics platforms built using Angular. Developed using TypeScript and SCSS, they can leverage the Angular framework and the broader Angular CLI toolchain for development, testing, and packaging. Additionally, RxJS is extensively used to manage asynchronous data streams, enabling efficient handling of user interactions, data loading, and reactive updates within the components. Despite their flexibility, these tools have already demonstrated their effectiveness in MobiDB, RepeatsDB, and RING 3.0 and 4.0 by successfully replacing previous visualization tools. This transition has reduced the overall complexity of these resources while improving both their user interface and user experience.

All components in the ngx-mol-viewers library are open-source and distributed under a CC-BY license. The codebase, along with practical examples and extensive documentation, is publicly available. The project team is committed to ongoing maintenance and actively welcomes feature requests and community feedback.

Each of the components below can be found on the NPM registry:

• ngx-structure-viewer
• ngx-features-viewer
• ngx-sequence-viewer

Moreover, the components come with complete and standardized API documentation pages that can be accessed using the following links, and from the library homepage:

• ngx-structure-viewer
• ngx-features-viewer
• ngx-sequence-viewer

Further information is available on the project’s GitHub repository: https://github.com/BioComputingUP/ngx-mol-viewers.

## Data Availability

Publicly available datasets were analyzed in this study. These data can be found here: https://github.com/BioComputingUP/ngx-mol-viewers.
